# Management of Refractory Orofacial Dyskinesia Caused by Anti-*N*-methyl-d-aspartate Receptor Encephalitis Using Botulinum Toxin

**DOI:** 10.3389/fneur.2018.00081

**Published:** 2018-02-22

**Authors:** Feixia Zheng, Xiuyun Ye, Xulai Shi, Neha Devi Poonit, Zhongdong Lin

**Affiliations:** ^1^Department of Pediatric Neurology, The Second Affiliated Hospital & Yuying Children’s Hospital of Wenzhou Medical University, Wenzhou, China; ^2^Department of Pediatrics, The Second Affiliated Hospital & Yuying Children’s Hospital of Wenzhou Medical University, Wenzhou, China

**Keywords:** anti-*N*-methyl-d-aspartate receptor encephalitis, dyskinesia, botulinum toxin, stereotypic movement disorder, vecuronium, rituximab, pediatrics

## Abstract

The use of botulinum neurotoxin serotype A (BoNT-A) injections for the treatment of orofacial dyskinesia secondary to anti-*N*-methyl-d-aspartate receptor (NMDAR) encephalitis is rarely reported. Here, we report a case of an urgent, successful management of severe orofacial dyskinesia in an 8-year-old girl with anti-NMDAR encephalitis using BoNT-A injection. The patient presented with *de novo* unilateral paroxysmal movement disorder progressing to generalized dystonia and repetitive orofacial dyskinesia. Diagnosis was confirmed by the presence of NMDAR antibodies in serum and cerebrospinal fluid. The orofacial dyskinesia worsened despite the aggressive use of first-line immunotherapy and second-line immunotherapy (rituximab), and resulted in a potentially fatal self-inflicted oral injury. We urgently attempted symptomatic management using BoNT-A injections in the masseter, and induced muscle paralysis using vecuronium. The patient’s severe orofacial dyskinesia was controlled. We observed the effects of the BoNT-A injections and a tapering off of the effects of vecuronium 10 days after the treatment. The movement disorder had improved significantly 4 weeks after the first administration of rituximab. The injection of BoNT-A into the masseter may be an effective treatment for medically refractory orofacial dyskinesia in pediatric patients with anti-NMDAR encephalitis. We propose that the use of BoNT-A injections should be considered early to avoid self-inflicted oral injury due to severe refractory orofacial dyskinesia in patients with anti-NMDAR encephalitis.

## Background

Anti-*N*-methyl-d-aspartate receptor (NMDAR) encephalitis is caused by autoimmune mechanisms associated with anti-NMDAR antibodies. This condition may appear in patients of all ages with or without tumors (usually an ovarian teratoma) (1–3). Most patients with anti-NMDAR encephalitis develop a multistage illness that progresses from psychosis, memory deficits, seizures, and language disintegration to a state of unresponsiveness with catatonic features often associated with movement disorders, and autonomic and breathing instability (2). Movement disorders, mostly in the form of orofacial and limb dyskinesias, are common features of anti-NMDAR encephalitis in young patients (3). Severe orofacial dyskinesia may result in self-inflicted oral injury, including lip and tongue injuries and broken teeth (4).

Data regarding the symptomatic treatment of movement disorders secondary to anti-NMDAR encephalitis before the action of immunotherapy are limited (5–10). To the best our knowledge, there have been no reports on the management of severe movement disorders in patients with anti-NMDAR encephalitis using botulinum neurotoxin serotype A (BoNT-A). Here, we report the case of a girl with anti-NMDAR encephalitis presenting with potentially fatal self-inflicted oral injury due to refractory orofacial stereotypy. Control of the patient’s condition required neuromuscular paralysis. The patient was successfully treated with an urgent injection of BoNT-A into the masseter.

## Case Presentation

An 8-year-old Chinese girl without any previous medical history presented with a new-onset paroxysmal movement disorder one month prior to hospitalization. The patient’s paroxysms, which comprised brief unilateral dystonic posturing of the left limb that lasted for minutes, had appeared after she was scolded by her parents. Routine video-electroencephalography (V-EEG) performed 1 week after the clinical onset (day 7) revealed no abnormal findings. On day 20, the patient presented with episodes of dystonia comprising wry neck and an irregular dystonic tremor in the limbs bilaterally, which was more prominent on the left side. Mild cognitive and memory deficits were also observed. 4 days prior to hospitalization, the patient’s mother had decided to seek medical assistance at our department, as the patient had developed insomnia and showed agitation, with occasional slurred speech.

On day 30 (the day of admission), a 2-h V-EEG revealed diffuse background slowing, which was more severe in the right hemisphere, and involuntary movements without paroxysmal activity or epileptiform discharges. This ruled out the possibility that the movements were associated with an epileptic pathophysiology. Cerebrospinal fluid analysis revealed pleocytosis (76 white blood cells μL^−1^) with normal protein and glucose levels. The immunological analysis revealed the presence of anti-NMDAR immunoglobulin G antibodies in the blood (titer: 1:32) and cerebrospinal fluid (titer: 1:10). Brain magnetic resonance imaging revealed no remarkable abnormalities. Serologic tests and polymerase chain reaction analysis of cerebrospinal fluid were performed for the detection of viruses, including the herpes simplex virus; however, no viral infection was detected. The results of tumor screening using thoracoabdominal computed tomography, pelvis magnetic resonance imaging, and assessment of serum tumor markers were negative.

**Figure 1:**
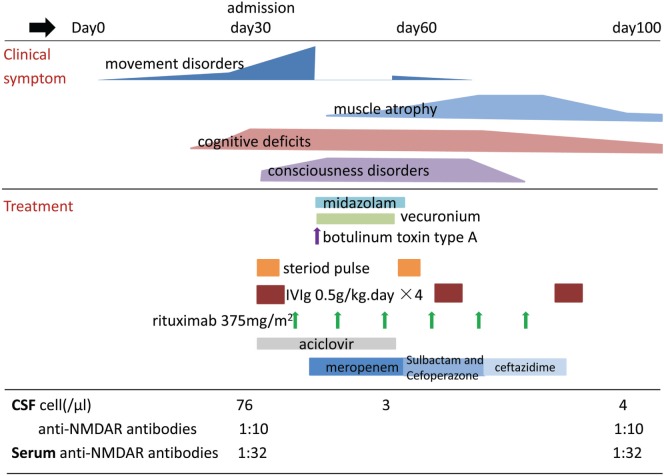
Schematic representation of the clinical course of the disease in our patient.

Consequently, the patient was diagnosed with anti-NMDAR encephalitis. We began concurrent treatment with immunoglobulin (0.5 g kg^−1^ daily for 4 days, three cycles) and methylprednisolone (20 mg kg^−1^ daily for 3 days, two cycles) on day 32. During the next few days, the patient developed coma and episodes of opisthotonus, chorea, limb myorhythmia, and orofacial stereotypy. We attempted to treat the patient using several oral anti-dystonia drugs, including clonazepam, baclofen, benzhexol hydrochloride, risperidone, and valproate, but they had minimal effects on her dyskinesia. Because there was no adequate response during the 10 days following treatment with first-line immunotherapy, we began treatment with intravenous rituximab (375 mg m^−2^ weekly for 6 weeks) on day 39. During this time, the patient developed self-inflicted oral injuries due to forceful stereotypic movements of the jaw and masticatory-like movements. The child frequently bit her tongue, lip, and buccal mucosa. After 2 days, she developed a high fever and neutrophilic leukocytosis in the blood (neutrophil count, 28.5 × 10^9^ cells L^−1^). *Burkholderia cepacia* was cultured from the patient’s oral secretion and was successfully treated using antibiotics. The self-inflicted oral injury progressed gradually from minor bruising to deep lacerations requiring suture repair, obvious bleeding, and two broken incisors. In order to control the stereotypic movements of the jaw, we began treatment with BoNT-A (50 U, 0.5 ml, injected into masseter on each side) and continuous infusion of midazolam (up to 0.25 mg kg^−1^ h^−1^) for muscle relaxation on day 44. Respiratory depression was noted following midazolam administration. Additionally, the patient’s orofacial stereotypy had led to potentially fatal oral bleeding. We were thus forced to start mechanical ventilation and continuous infusion of vecuronium (0.07–0.08 mg kg^−1^ h^−1^), to control the patient’s injurious movements. This resulted in complete control of all movement disorders, allowing surgical debridement and suturing of the tongue and lip. On day 54 (10 days after injection of BoNT-A), the intensity and frequency of the orofacial stereotypy was obviously reduced in a therapeutic window of a few hours after withholding the intravenous vecuronium drip, but opisthotonus, chorea, and limb myorhythmia had still not improved. Vecuronium administration was tapered following this observation and was discontinued after 12 h. On day 58 (3 weeks after the first administration of rituximab), with the tapering of vecuronium and midazolam, hypoventilation was markedly improved and artificial ventilation was discontinued. On day 67 (4 weeks after the first administration of rituximab), B cells were effectively depleted, and the effect of immunotherapy appeared. Opisthotonus, chorea, limb myorhythmia, and consciousness disturbance improved gradually. However, the patient was unable to move her legs, had a left drop foot, and could not eat or sit unassisted. On day 100, she regained the ability to perform daily activities with minimal need for assistance with walking and was discharged (Figure 1). On day 160, muscle strength returned to normal. Eighteen months after disease onset, no abnormal findings were observed on V-EEG. At this time, the patient showed almost complete mental recovery and attended regular school in an age-appropriate class. During a follow-up of over 2 years, the antibody titers in the patient’s serum remained stable and no tumor was identified.

## Discussion

Movement disorders are the second most frequent initial symptoms in children (aged <12 years) with anti-NMDAR encephalitis (3), and have been reported in 84% of the cases. These movement disorders include orofacial dyskinesia; choreoathetosis; chorea; limb, trunk, and facial dystonia; tremor; ataxia; opsoclonus-myoclonus; and myoclonus (1, 2, 10). Stereotyped movements and orofacial dyskinesia are the most common movement disorders, occurring in 85 and 45% of the patients, respectively (1). Orofacial dyskinesia has the most distinctive clinical features, including grimacing, forceful jaw opening and closing, and masticatory-like movements (2, 4, 10, 11). The complexity of limb and orofacial movements in patients with anti-NMDAR encephalitis is likely explained by the disinhibition of a brainstem central pattern generator, involving dopaminergic pathways, which are inhibited by the GABAergic system under normal conditions (12).

The treatment of secondary movement disorders relies on the treatment of the underlying pathologic process. Patients with anti-NMDAR encephalitis usually respond to immunotherapy (corticosteroids, intravenous immunoglobulin, or plasma exchange) and tumor removal. Second-line immunotherapy (rituximab and/or cyclophosphamide) has been shown to significantly improve outcomes in patients who do not respond to first-line therapy and to decrease the frequency of relapses (2, 3). Children are generally treated with only one second-line immunotherapy agent (usually rituximab) (13). There are no clear consensus criteria regarding when to initiate second-line immunotherapy for patients with anti-NMDAR encephalitis. Currently, second-line immunotherapy is usually started 10–14 days after non-response to first-line immunotherapy; however, it should be considered earlier in patients without tumors or for those with delayed diagnosis (2, 14). Movement disorders due to anti-NMDAR encephalitis usually improve with immunosuppressive therapy (10, 15). In the current case, the movement disorder was significantly improved when B cells were depleted using rituximab. This finding was consistent with those of previous studies (10, 15).

In a retrospective series conducted in pediatric patients by Mohammad et al. (8), 85% of the children were treated for movement disorders, which were often difficult to control, making movement disorder the third most frequent symptom treated in children with anti-NMDAR encephalitis, following agitation and seizures. Symptomatic treatment is often initiated to reduce the risk of injury or rhabdomyolysis, to facilitate patient care, and to reduce parental and patient distress (8). There are no recommendations regarding the treatment of severe movement disorder before immunotherapy has had time to take effect. Treatment options for childhood dystonia include several oral pharmaceutical agents, botulinum toxin injection, and deep brain stimulation therapy (16). Medications used for the treatment of primary dystonia may also be used for the treatment of secondary dystonia (16, 17).

Almost invariably, dyskinesia secondary to anti-NMDAR encephalitis requires control using relaxation or deep sedation with a combination of drugs (18). In the retrospective series by Mohammad et al. (8), sedatives such as clonidine, benzodiazepines, and some anticonvulsants (phenobarbital, sodium valproate, and carbamazepine) appeared to be beneficial in managing movement disorders. In the same study, levodopa and central anticholinergics (benztropine and benzhexol) had moderate benefits, while baclofen and amantadine had no effect. In another case series, tetrabenazine, a monoamine depletor of the central nervous system, was reported effective for the treatment of dyskinesia in one patient (10). Antipsychotics, such as haloperidol, appear to have a vulnerability to antipsychotic-induced adverse events, with extrapyramidal symptoms in children with anti-NMDAR, which may worsen dyskinesia (8). Several case reports, similar to the present case, indicate that dopamine blockers, antiepileptic drugs, and benzodiazepines are ineffective for the treatment of dyskinesia in anti-NMDAR encephalitis (6, 7, 9). The reason might be that the abnormal movements seen in this disease are not purely caused by basal ganglia dysfunction.

Life-threatening movement disorders often require prompt, complex, and aggressive interventions. Stronger sedation and muscle paralysis are most likely to achieve prompt resolution of movement disorders (16, 17, 19). Midazolam is usually chosen as the treatment of choice, because of its rapid onset of action and muscle relaxant effect. A selective modulator of gamma-aminobutyric acid A receptors, i.e., propofol, may be added for patients who require deeper sedation. Non-depolarizing muscle paralyzing agents and barbiturates are used for patients with refractory movement disorders (16, 17). Several adult case reports and a case series have described the treatment of severe dyskinesia in patients with anti-NMDAR encephalitis (Table 1). Isoflurane has been reported to be effective in controlling intolerable dyskinesia in a woman with anti-NMDAR encephalitis, although clinical improvement was not achieved before 4 months of continuous treatment (6). In other similar cases, severe dyskinesia was fully controlled after the administration of the first dose of tramadol, in a 10-week course of treatment (7). Electroconvulsive therapy was successfully used for the treatment of refractory dyskinesia, which required continuous infusion of a neuromuscular blocker, after long-lasting treatment with various forms of immunotherapy had failed (9). Ketamine has also been described as a useful treatment for severe dyskinesia in a young woman with anti-NMDAR encephalitis (5), but reported ineffective in another two cases (20).

**Table 1:** Treatment protocols for severe dyskinesia in patients with anti-*N*-methyl-d-aspartate receptor (NMDAR) encephalitis.

Reference	Age (years)/sex	Anti-NMDAR antibody titers	Agent	Dose	Onset time	Duration	Side effects	Ineffective drugs used
Gumbinger et al. (6)	38/F	CSF: 1:320, serum: 1:3200	Isoflurane	MAC: started at 1.90, reduced to <0.3	Immediately	4 months	No	Tiapride, biperiden, ketamine, midazolam, and propofol

Sunwoo et al. (9)	27/F	CSF: +, serum: +	ECT, Cisatracurium	192–432 mC with an 800-mA current; 2 sessions/week 4 µg kg^−1^ min^−1^, tapered off after each session of ECT	After 3 sessions Immediately	6 weeks3 weeks	Not mentioned	AED, benzodiazepine, and olanzapine

Seifi and Kitchen (7)	23/F	CSF: +	Tramadol	100 mg, Q6h	After the first dose	10 weeks	No	Ketamine, lorazepam, and dextromethorphan

MacMahon et al. (5)	21/F	CSF: +, serum: −	Ketamine	20 mg/h	After a few hours	2 weeks	Not mentioned	Propofol, alfentanil, benzodiazepine, clonidine, dexmedetomidine, and risperidone

*AED, antiepileptic drug; ECT, electroconvulsive therapy; MAC, minimal alveolar concentration; mC, millicoulomb; Q6h, every 6 h; +, positive; −, negative*.

Further, BoNT-A has been used to achieve therapeutic benefit in patients with focal dystonia (e.g., focal tics, oromandibular dystonia, and other hyperkinetic disorders) in conjunction with drug therapy (21, 22). After injection into muscle, the BoNT-A single-chain polypeptide has little potency. Cleavage of BoNT-A by trypsin into its heavy and light chain components, which are subsequently linked by a disulfide bond, produces the active toxin molecule (23). The BoNT-A molecule is internalized into presynaptic nerve terminals via the binding of its heavy chain to a cell-surface receptor, which consists of a ganglioside and a cell-surface protein (24). Following entry into the nerve terminal, the light chain is internalized into a vesicle, where it binds to the highly specific soluble *N*-ethylmaleimide-sensitive factor attachment protein receptor complex. Binding to the complex enables the light chain of BoNT-A to cleave synaptosomal-associated proteins of 25 kDa, which mediate the docking and fusion of neurotransmitter-containing vesicles to the presynaptic membrane (23, 24). Cleavage of synaptosomal-associated proteins of 25 kDa prevents exocytosis of neurotransmitters from the presynaptic terminal. Preventing the release of neurotransmitters can cause muscle paralysis (23). Moreover, BoNT-A can irreversibly block the release of acetylcholine at the neuromuscular junctions in the injected muscle, and weakens related dystonic muscles (21, 22). The effects of BoNT-A injections typically appear within 1–2 weeks, and peak at around 4–6 weeks, and start wearing off around 2.5–3 months (16, 22). The injection of BoNT-A is an effective and well-tolerated treatment, with long-term benefits against movement disorders (22). In the current case, immunotherapy, sedative medication, and BoNT-A were administered at the same time. The improvement of refractory orofacial dyskinesia was observed at the time when the effect of BoNT-A injections typically appears, and 2 weeks before clinical improvement, including the improvement of other dyskinesia, when the immunotherapy exerted its effects. The improvement is not likely to be due to midazolam because of its rapid onset of action. Therefore, we concluded that the improvement of orofacial stereotypy in our patient was likely to be the effect of BoNT-A injection, before immunotherapy could produce results. Our study is the first report of urgent and successful symptomatic management of refractory dyskinesia secondary to anti-NMDAR encephalitis, using BoNT-A injection. Compared to other reported treatment protocols described above, BoNT-A injection is more convenient and safe, but has a longer delay before the onset of effects (22). Thus, we propose that early administration of BoNT-A may be helpful in avoiding severe self-inflicted oral injuries and the need for more aggressive interventions.

Orofacial dyskinesia, which was the major concern for the patient in this study, was so severe that continuous infusion of a neuromuscular blocker was required to control self-harming activities, which were not predictable at the early stages of the disease. The injection of BoNT-A into the masseter successfully controlled the orofacial dyskinesia in our patient. Nevertheless, the suitable time to begin BoNT-A therapy for avoiding severe self-inflicted oral injury, and the need for more aggressive interventions, in patients with anti-NMDAR encephalitis is as yet unclear. Further studies are required to identify the early warning signs for severe orofacial dyskinesia in patients with anti-NMDAR encephalitis, and the appropriate timing for the initiation of BoNT-A therapy.

## Conclusion

Orofacial dyskinesia secondary to anti-NMDAR encephalitis may result in severe self-inflicted oral injury. However, BoNT-A therapy may be used to successfully control refractory orofacial dyskinesia in patients with anti-NMDAR encephalitis, while waiting for immunotherapy to aid in recovery. Therefore, to avoid severe self-inflicted oral injury, early aggressive injection of BoNT-A into the masseter should be considered as a treatment option for medically refractory orofacial dyskinesia in patients with anti-NMDAR encephalitis. Further studies on the early warning signs of severe orofacial dyskinesia in patients with anti-NMDAR encephalitis, and the appropriate timing for the initiation of BoNT-A therapy, are warranted.

## Informed Consent

Written informed consent was obtained from the patient’s mother, in accordance with the Declaration of Helsinki, for the publication of this case report.

## Ethics Statement

Ethical approval was not required for this study, as per the national guidelines.

## Author Contributions

FZ, XS, XY, and ZL treated the patient; FZ and ZL contributed to the conception of the study; FZ drafted the initial manuscript; ZL critically reviewed the manuscript; and NP reviewed and revised the manuscript. All authors have read and approved the final manuscript.

## Conflict of Interest Statement

The authors declare that the research was conducted in the absence of any commercial or financial relationships that could be construed as a potential conflict of interest.

## Abbreviations

BoNT-A, botulinum neurotoxin serotype A; NMDAR, *N*-methyl-d-aspartate receptor; V-EEG, video-electroencephalography.
